# Supplementary material for: Excess death estimates from multiverse analysis in 2009–2021
Source: Eur J Epidemiol. 2023 Apr 12;38(11):1129–39. doi: 10.1007/s10654-023-00998-2 (PMC10090741; doi:10.1007/s10654-023-00998-2)
Supplement: Supplementary file 1 — Supplementary file1 (PDF 7716 KB) [file 10654_2023_998_MOESM1_ESM.pdf]

13 SUPPLEMENTARY TABLES & FIGURES

Excess death estimates from multiverse analysis in 2009-2021

Michael Levitt[1], Francesco Zonta[2], John P.A. Ioannidis[3]

[1]Department of Structural Biology, Stanford University, Stanford, CA 94305, USA

[2]Shanghai Institute for Advanced Immunochemical Studies, ShanghaiTech University, Shanghai 201210, China

[3]Departments of Medicine, of Epidemiology and Population Health, of Biomedical Data Science, and of Statistics, and Meta-Research Innovation Center at Stanford (METRICS), Stanford University, Stanford, CA 94305, USA

Correspondence mail: jioannid@stanford.edu

Figure S1: Comparing the multiverse relative excess death, p%, calculated with the 66 reference periods with p% for the single reference period (years 2017 to 2019) published before (6). The multiverse p% values are calculated with dw3 weighting and no weighting (dw0). The fitted linear trends show that dw3 p% values are much closer to 2017-19 p% value with high correlation of 0.997 and average shift of -1.1 percentage points. For the dw0 fit, the correlation is still high at 0.971 but the data is more scattered and the values shift downward by 4.6 percentage points. The gray line is diagonal and shows where y=x

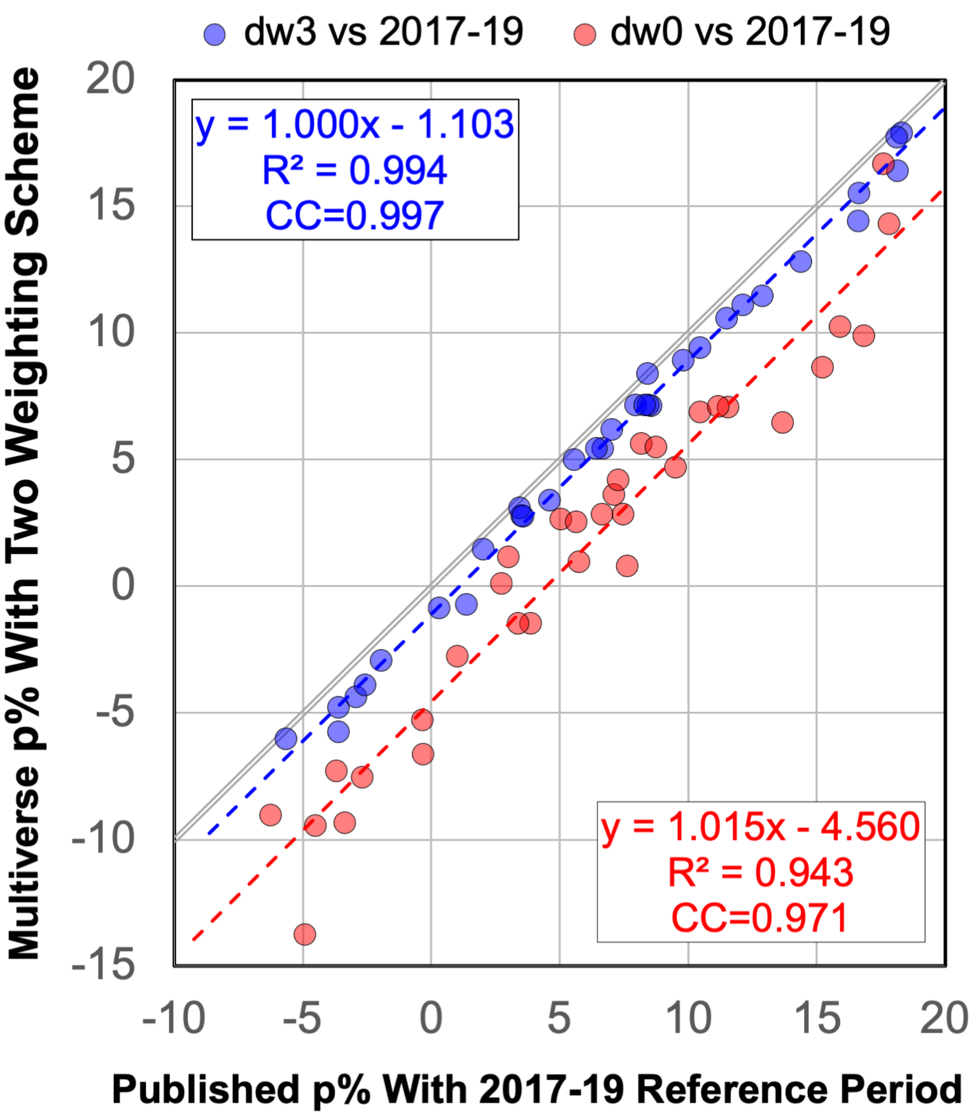

Figure S2A: Distribution of the rank of country relative excess death estimates (highest to lowest) in the one-year projected period 2021 for the 33 countries as calculated for each of the 66 different reference baseline year sets. The countries are ordered by decreasing average rank (column 3); the standard deviation of the rank is given in column 4. The country rank is ambiguous for the seven countries between Germany and Netherlands. High- and low-ranking countries are less ambiguous. For 22 countries the diagonal entry occurs most often.

Country	Rank		Number of rank occurrences in sort from highest to lowest p% for single year 2021																																
	AVE	SD	1	2	3	4	5	6	7	8	9	10	11	12	13	14	15	16	17	18	19	20	21	22	23	24	25	26	27	28	29	30	31	32	33
Slovakia	1.33	0.56	47	16	3																														
Poland	2.42	0.55		40	24	2																													
United States	2.98	1.67	19	8	13	15	4	6	1																										
Latvia	3.58	0.55		2	24	40																													
Czechia	5.26	0.84			1	6	40	15	2	2																									
Lithuania	5.74	0.93			1	3	19	36	4	2	1																								
Croatia	7.45	0.96					3	2	31	24	5		1																						
Hungary	7.73	0.85						4	21	32	7	2																							
Chile	9.48	1.18						1	7	3	11	36	8																						
Greece	9.48	0.89						1			42	11	12																						
Estonia	10.86	1.69						1		3		17	40	1		1		1	1	1															
Germany	14.02	2.10												18	19	8	6	5	5	2	1	2													
United	14.50	2.23											3	13	13	7	4	9	11	4	2														
Austria	14.74	2.72												24	5	6	7	4	4	8	6	1	1												
Italy	15.20	1.84											2	3	4	13	17	15	5	4	1	2													
Portugal	15.23	1.76												3	6	18	11	12	9	6			1												
Slovenia	16.18	2.75												4	17	4	4	3	3	9	18	4													
Netherlands	16.35	1.58													2	8	14	7	12	22	1														
France	17.92	1.37														1	1	10	15	6	29	4													
Canada	20.15	0.97															1			2	3	41	16	3											
Israel	20.92	1.34															1		1	2	3	9	25	24	1										
Spain	21.56	0.84																			2	3	22	35	3	1									
Finland	23.56	0.84																						1	37	22	2	4							
Luxembourg	24.68	1.78																						3	16	23	4	3	11	6					
Belgium	25.26	1.19																							5	10	23	23	3		2				
Switzerland	26.09	1.28																							1	7	13	17	22	5		1			
Denmark	26.95	1.94																								1	19	10	17	5	6	3	3	2	
Iceland	28.39	2.58																					1		3	1	3	7	7	10	12	3	14	3	2
New Zealand	28.56	1.45																								1	1	2	5	24	22	6	2	2	1
Australia	29.71	0.83																									1			2	14	45	4		
Norway	29.94	1.27																											1	13	10	7	35		
Sweden	31.86	0.60																												1			7	56	2
South Korea	32.88	0.48																														1	1	3	61

Figure S2B: Distribution of the rank of country excess death estimates (from highest to lowest) in the three-year projected period 2019+2020+2021 expressed as a percentage of the expected deaths for the 33 countries as calculated for each of the 66 different reference baseline year sets. The countries are ordered by decreasing average rank (column 3); the standard deviation of the rank is given in column 4. High- and low-ranking countries have consistent ranks but those lying in between are more ambiguous. 18 on diagonal

Country	Rank		Number of rank occurrences in sort from highest to lowest p% for 3-year projected period 2019+2020+2021																																
	AVE	SD	1	2	3	4	5	6	7	8	9	10	11	12	13	14	15	16	17	18	19	20	21	22	23	24	25	26	27	28	29	30	31	32	33
United States	1.27	0.54	50	15		1																													
Poland	1.77	0.42	15	51																															
Czechia	3.41	1.06			52	8	4			1	1																								
Slovakia	6.00	3.39	1		2	35	5	6	2	1	4		3	3		1	2		1																
Greece	6.20	2.74			3	12	22	9	7	4	2	1	2	1	1	1				1															
Hungary	7.08	1.44					13	8	20	15	7	2	1																						
Lithuania	7.21	2.14			2	4	7	15	8	15	6	4	2	2	1																				
Latvia	7.85	2.43			2		4	15	13	11	6	8	3	2		1				1															
Croatia	8.68	2.27			1	4	1	2	7	11	21	11	3	1	2	1		1																	
Italy	9.85	2.68			2	1	1	3	6	3	7	16	11	10	3	1		1		1															
Chile	10.95	4.11			2		7	5	1	2	4	7	10	7	4	4	2	4	2	3	1	1													
United Kingdom	12.29	3.19				1	2	2	1	3	3	2	11	6	11	3	11	8	1		1														
Slovenia	13.44	2.89									2	5	12	15	8	5	2	1	6	5	5														
France	13.88	2.68							1		1	4	3	12	10	15	4	3	6	3	1	3													
Portugal	15.65	2.31									2	1			6	7	19	7	10	8	3	2	1												
Spain	15.88	3.09						1				3	1	3	8	2	8	10	13	4	7	3		2	1										
Netherlands	16.55	2.40											2	1	1	9	11	10	6	14	6	3	2			1									
Austria	16.70	2.72											2	2	10	3	4	7	7	7	15	7	2												
Germany	18.15	2.46										1				3	2	9	11	11	15	5	3	1	3	2									
Estonia	20.64	4.13										1			1	9	1	2	1	2	3	6	6	8	8	10	4	1	1	1	1				
Canada	20.68	2.13												1				2	1	2	5	26	4	13	5	7									
Belgium	20.88	1.16																1		2	3	8	39	10	2	1									
Israel	22.52	1.62														1				1		1	5	23	22	9	3	1							
Switzerland	23.71	1.23																				1	2	6	18	21	16	1	1						
Luxembourg	24.47	1.70																	1		1		2	2	7	9	31	10	3						
Finland	26.38	0.62																									2	40	21	3					
Iceland	27.64	2.79																		1				1		4	9	8	12	6	6	9	2	7	1
Denmark	28.52	1.71																										1	24	16	9	4	5	7	
New Zealand	28.70	1.53																								1	1	2		31	18	7	1	3	2
Sweden	29.12	1.23																								1		1	4	8	25	22	5		
Australia	30.80	1.00																										1		1	2	13	38	10	1
Norway	31.27	0.95																													4	11	14	37	
South Korea	32.88	0.56																													1		1	2	62

Figure S2C: Distribution of the rank of country excess death estimates (from highest to lowest) in the four-year projected period 2018+2019+2020+2021 expressed as a percentage of the expected deaths for the 33 countries as calculated for each of the 66 different reference baseline year sets. The countries are ordered by decreasing average rank (column 3); the standard deviation of the rank is given in column 4. High- and low-ranking countries have consistent ranks but those lying in between are more ambiguous. The most common rank occurrence in on the diagonal 17 times.

Country	Rank		Rank in Sort from Highest p% to Lowest p% For Four Years 2018, 2019, 2020 & 2021																																
	AVE	SD	1	2	3	4	5	6	7	8	9	10	11	12	13	14	15	16	17	18	19	20	21	22	23	24	25	26	27	28	29	30	31	32	33
**United States**	1.30	0.63	49	16			1																												
**Poland**	1.77	0.45	16	49	1																														
**Czechia**	3.91	1.74			44	7	7	1	5			1		1																					
**Hungary**	6.27	1.74				11	13	17	11	4	7	2	1																						
**Latvia**	6.89	2.84			1	9	19	9	7	4	6	4	1	2	2	1			1																
**Greece**	7.50	3.39			3	3	11	15	12	7	3	2	1	3	2		1	1	1				1												
**Slovakia**	7.53	4.84	1	1	1	27	5	3	6	1	3	2	3	1		1	3	4		2	1	1													
**Croatia**	9.05	3.04			2	4		1	5	23	11	6	4	1	1	3	4					1													
**Lithuania**	9.18	2.97			2	1	4	6	6	5	15	7	8	3	3	4			2																
**Italy**	10.21	3.31			2	2		1	7	6	9	13	5	8	5	2	3	1				1	1												
**United Kingdom**	11.02	4.66			8	1	2	4	2	3	2	2	12	2	3	6	4	9	4		2														
**France**	13.12	3.55					1	2		1	4	7	5	16	5	3	5	3	4	4	2	4													
**Chile**	14.17	5.38			2		3	6		2	2	2	6	3	2	1	2	2	5	11	7	6	4												
**Slovenia**	14.62	3.75								3	1	8	7	6	4	2	2	5	12	5	3	6	2												
**Portugal**	14.82	2.82						1	1	2			2	3	9	8	10	13	9	4	3			1											
**Germany**	14.83	3.87							2	2		4	3	2	13	12	5	3	4	4	4	2	2	1		3									
**Spain**	15.48	3.79				1			2	1		3	2	4	4	5	7	10	7	9	2	4	2	1	2										
**Netherlands**	15.56	3.03								1	1	2	2	4	5	7	13	4	10	7	5		4		1										
**Austria**	17.08	3.09									2	1	2	2	4	4	2	6	3	6	22	10	2												
**Canada**	19.55	2.97											1	1	2	1	1	3	4	8	6	16	5	5	9	4									
**Belgium**	20.86	1.48															1			5	3	6	35	10	5		1								
**Estonia**	21.11	4.86								1			1	3	1	4	3	1		1	3	4	4	13	5	6	8	2	2	1	1	1	1		
**Israel**	22.98	1.72														1					1	1		24	9	24	4	1	1						
**Luxembourg**	23.55	2.71													1	1		1			1	3	2	5	15	6	19	8	4						
**Switzerland**	24.00	1.34																				1	1	5	18	13	24	1	3						
**Finland**	26.32	0.68																								1	2	41	19	3					
**Iceland**	27.59	3.54												1									1	1	2	7	6	5	8	8	5	6	7	7	2
**Denmark**	28.09	1.68																									2	4	25	13	12	2	3	5	
**New Zealand**	28.67	1.63																								2		2	2	29	18	7	1	2	3
**Sweden**	29.08	1.60																			1							1	2	12	19	28	3		
**Norway**	30.98	1.04																													7	15	16	28	
**Australia**	31.08	1.02																										1			3	7	33	21	1
**South Korea**	32.83	0.62																													1		2	3	60

Figure S3: Showing how the ranking of the relative excess death over the years between 2009 and 2021 for the 66 different reference periods is almost independent on the choice of reference set. There are 132 rankings of the 33 countries and 4 averaging periods. Of these 132 rankings, 67 have identical rankings for all reference years. Below we show the two cases for each averaging period with rankings that differ most from the average rank ordering. When not zero, the standard deviation in a ranking is approximately 0.5. Error is defined as the sum of the off-diagonal occurrence multiplied by the distance from the diagonal. Only in one case is the diagonal element smaller than an off-diagonal element: Year 2013 for USA averaged over 2 years and the standard deviation then reaches 0.86. This occurs because the p% values for the USA are flat before 2020 (see Fig. 2) and hard to distinguish.

Average over One Year

ESP	Mean	SD	p%	1	2	3	4	5	6	7	8	9	10	11	12	13	Error
2020	1	0	-6.59	66	0	0	0	0	0	0	0	0	0	0	0	0	0
2009	2	0	0.00	0	66	0	0	0	0	0	0	0	0	0	0	0	0
2012	3	0	1.93	0	0	66	0	0	0	0	0	0	0	0	0	0	0
2010	4	0	6.58	0	0	0	66	0	0	0	0	0	0	0	0	0	0
2015	5	0	-2.13	0	0	0	0	66	0	0	0	0	0	0	0	0	0
2011	6	0	3.15	0	0	0	0	0	66	0	0	0	0	0	0	0	0
2017	7	0	-2.48	0	0	0	0	0	0	66	0	0	0	0	0	0	0
2013	8.38	0.49	3.22	0	0	0	0	0	0	0	41	25	0	0	0	0	25
2021	8.62	0.49	8.70	0	0	0	0	0	0	0	25	41	0	0	0	0	50
2014	10	0	-1.46	0	0	0	0	0	0	0	0	0	66	0	0	0	50
2016	11.41	0.49	2.32	0	0	0	0	0	0	0	0	0	0	39	27	0	77
2018	11.59	0.49	-0.81	0	0	0	0	0	0	0	0	0	0	27	39	0	104
2019	13	0	-2.50	0	0	0	0	0	0	0	0	0	0	0	0	66	104

HRV	Mean	SD	p%	1	2	3	4	5	6	7	8	9	10	11	12	13	Error
2021	1.0	0.0	2.03	66	0	0	0	0	0	0	0	0	0	0	0	0	0
2009	2.0	0.0	0.00	0	66	0	0	0	0	0	0	0	0	0	0	0	0
2010	3.0	0.0	8.97	0	0	66	0	0	0	0	0	0	0	0	0	0	0
2011	4.0	0.0	7.17	0	0	0	66	0	0	0	0	0	0	0	0	0	0
2012	5.0	0.0	3.08	0	0	0	0	66	0	0	0	0	0	0	0	0	0
2015	6.0	0.0	-2.15	0	0	0	0	0	66	0	0	0	0	0	0	0	0
2020	7.0	0.0	-6.86	0	0	0	0	0	0	66	0	0	0	0	0	0	0
2017	8.30	0.46	-3.84	0	0	0	0	0	0	0	46	20	0	0	0	0	20
2013	8.70	0.46	2.62	0	0	0	0	0	0	0	20	46	0	0	0	0	40
2014	10	0	-1.70	0	0	0	0	0	0	0	0	0	66	0	0	0	40
2016	11.32	0.47	2.41	0	0	0	0	0	0	0	0	0	0	45	21	0	61
2018	11.68	0.47	-1.63	0	0	0	0	0	0	0	0	0	0	21	45	0	82
2019	13	0	-3.87	0	0	0	0	0	0	0	0	0	0	0	0	66	82

Average over Two Years

CAN	Mean	SD	p%	2	3	4	5	6	7	8	9	10	11	12	13	Error
2010	1	0	0.00	66	0	0	0	0	0	0	0	0	0	0	0	0
2011	2	0	5.31	0	66	0	0	0	0	0	0	0	0	0	0	0
2012	3	0	2.73	0	0	66	0	0	0	0	0	0	0	0	0	0
2014	4.24	0.43	0.12	0	0	0	50	16	0	0	0	0	0	0	0	16
2013	5.38	0.49	1.18	0	0	0	0	41	25	0	0	0	0	0	0	41
2021	5.38	0.85	-1.94	0	0	0	16	9	41	0	0	0	0	0	0	82
2015	7	0	0.17	0	0	0	0	0	0	66	0	0	0	0	0	82
2018	8	0	-1.72	0	0	0	0	0	0	0	66	0	0	0	0	82
2017	9	0	-1.78	0	0	0	0	0	0	0	0	66	0	0	0	82
2016	10	0	-0.35	0	0	0	0	0	0	0	0	0	66	0	0	82
2020	11	0	-3.23	0	0	0	0	0	0	0	0	0	0	66	0	82
2019	12	0	-1.53	0	0	0	0	0	0	0	0	0	0	0	66	82

USA	Mean	SD	p%	2	3	4	5	6	7	8	9	10	11	12	13	Error
2021	1	0	7.05	66	0	0	0	0	0	0	0	0	0	0	0	0
2020	2	0	-1.30	0	66	0	0	0	0	0	0	0	0	0	0	0
2010	3	0	0.00	0	0	66	0	0	0	0	0	0	0	0	0	0
2011	4	0	1.27	0	0	0	66	0	0	0	0	0	0	0	0	0
2012	5	0	0.91	0	0	0	0	66	0	0	0	0	0	0	0	0
2017	6.21	0.41	-0.10	0	0	0	0	0	52	14	0	0	0	0	0	14
2013	7.32	0.86	0.46	0	0	0	0	0	14	20	29	3	0	0	0	63
2016	7.52	0.50	-0.43	0	0	0	0	0	0	32	34	0	0	0	0	95
2018	8.95	0.21	-0.03	0	0	0	0	0	0	0	3	63	0	0	0	98
2015	10	0	-0.70	0	0	0	0	0	0	0	0	0	66	0	0	98
2014	11	0	-0.10	0	0	0	0	0	0	0	0	0	0	66	0	98
2019	12	0	-0.25	0	0	0	0	0	0	0	0	0	0	0	66	98

Average over Three Years

ISL	Mean	SD	p%	3	4	5	6	7	8	9	10	11	12	13	Error
2011	1	0	0.00	66	0	0	0	0	0	0	0	0	0	0	0
2017	2.33	0.53	0.07	0	46	18	2	0	0	0	0	0	0	0	22
2012	2.71	0.49	3.67	0	20	45	1	0	0	0	0	0	0	0	23
2015	4.44	0.53	-0.98	0	0	1	35	30	0	0	0	0	0	0	54
2013	4.52	0.56	0.58	0	0	2	28	36	0	0	0	0	0	0	86
2016	6	0	0.38	0	0	0	0	0	66	0	0	0	0	0	86
2018	7.35	0.48	0.60	0	0	0	0	0	0	43	23	0	0	0	109
2014	7.65	0.48	0.38	0	0	0	0	0	0	23	43	0	0	0	132
2019	9	0	-0.97	0	0	0	0	0	0	0	0	66	0	0	132
2020	10	0	-3.71	0	0	0	0	0	0	0	0	0	66	0	132
2021	11	0	-5.53	0	0	0	0	0	0	0	0	0	0	66	132

USA	Mean	SD	p%	3	4	5	6	7	8	9	10	11	12	13	Error
2021	1	0	4.42	66	0	0	0	0	0	0	0	0	0	0	0
2020	2	0	-0.74	0	66	0	0	0	0	0	0	0	0	0	0
2011	3	0	0.00	0	0	66	0	0	0	0	0	0	0	0	0
2012	4	0	1.15	0	0	0	66	0	0	0	0	0	0	0	0
2013	5	0	0.60	0	0	0	0	66	0	0	0	0	0	0	0
2017	6	0	-0.46	0	0	0	0	0	66	0	0	0	0	0	0
2018	7.38	0.52	0.08	0	0	0	0	0	0	42	23	1	0	0	25
2015	7.64	0.48	-0.47	0	0	0	0	0	0	24	42	0	0	0	49
2016	9.39	0.49	-0.36	0	0	0	0	0	0	0	0	40	26	0	75
2014	9.59	0.52	0.23	0	0	0	0	0	0	0	1	25	40	0	102
2019	11	0	-0.33	0	0	0	0	0	0	0	0	0	0	66	102

Average over Four Years

AUT	Mean	SD	p%	4	5	6	7	8	9	10	11	12	13	Error
2012	1	0	0.00	66	0	0	0	0	0	0	0	0	0	0
2013	2	0	4.31	0	66	0	0	0	0	0	0	0	0	0
2014	3	0	3.27	0	0	66	0	0	0	0	0	0	0	0
2015	4	0	1.79	0	0	0	66	0	0	0	0	0	0	0
2016	5.39	0.49	1.33	0	0	0	0	40	26	0	0	0	0	26
2021	5.61	0.49	-2.39	0	0	0	0	26	40	0	0	0	0	52
2017	7.39	0.49	-0.83	0	0	0	0	0	0	40	26	0	0	78
2020	7.61	0.49	-4.39	0	0	0	0	0	0	26	40	0	0	104
2018	9	0	-2.35	0	0	0	0	0	0	0	0	66	0	104
2019	10	0	-2.99	0	0	0	0	0	0	0	0	0	66	104

USA	Mean	SD	p%	4	5	6	7	8	9	10	11	12	13	Error
2021	1	0	3.45	66	0	0	0	0	0	0	0	0	0	0
2020	2	0	-0.68	0	66	0	0	0	0	0	0	0	0	0
2012	3	0	0.00	0	0	66	0	0	0	0	0	0	0	0
2013	4	0	0.86	0	0	0	66	0	0	0	0	0	0	0
2014	5.32	0.47	0.40	0	0	0	0	45	21	0	0	0	0	21
2018	5.73	0.54	-0.23	0	0	0	0	21	42	3	0	0	0	45
2017	7.12	0.33	-0.39	0	0	0	0	0	0	58	8	0	0	53
2015	7.83	0.48	-0.13	0	0	0	0	0	3	5	58	0	0	64
2016	9	0	-0.27	0	0	0	0	0	0	0	0	66	0	64
2019	10	0	-0.18	0	0	0	0	0	0	0	0	0	66	64

Figure S4A: Variation with year from 2009 to 2021 of the relative excess death, p%, calculated for single years. The salmon shading marks the range of all 66 reference periods, the purple shading marks the range between the first and third quartile and the black line shows the median for the reference periods. A year with a low p% value is often followed by a year with a high value. These fluctuations are averaged out with longer projected periods.

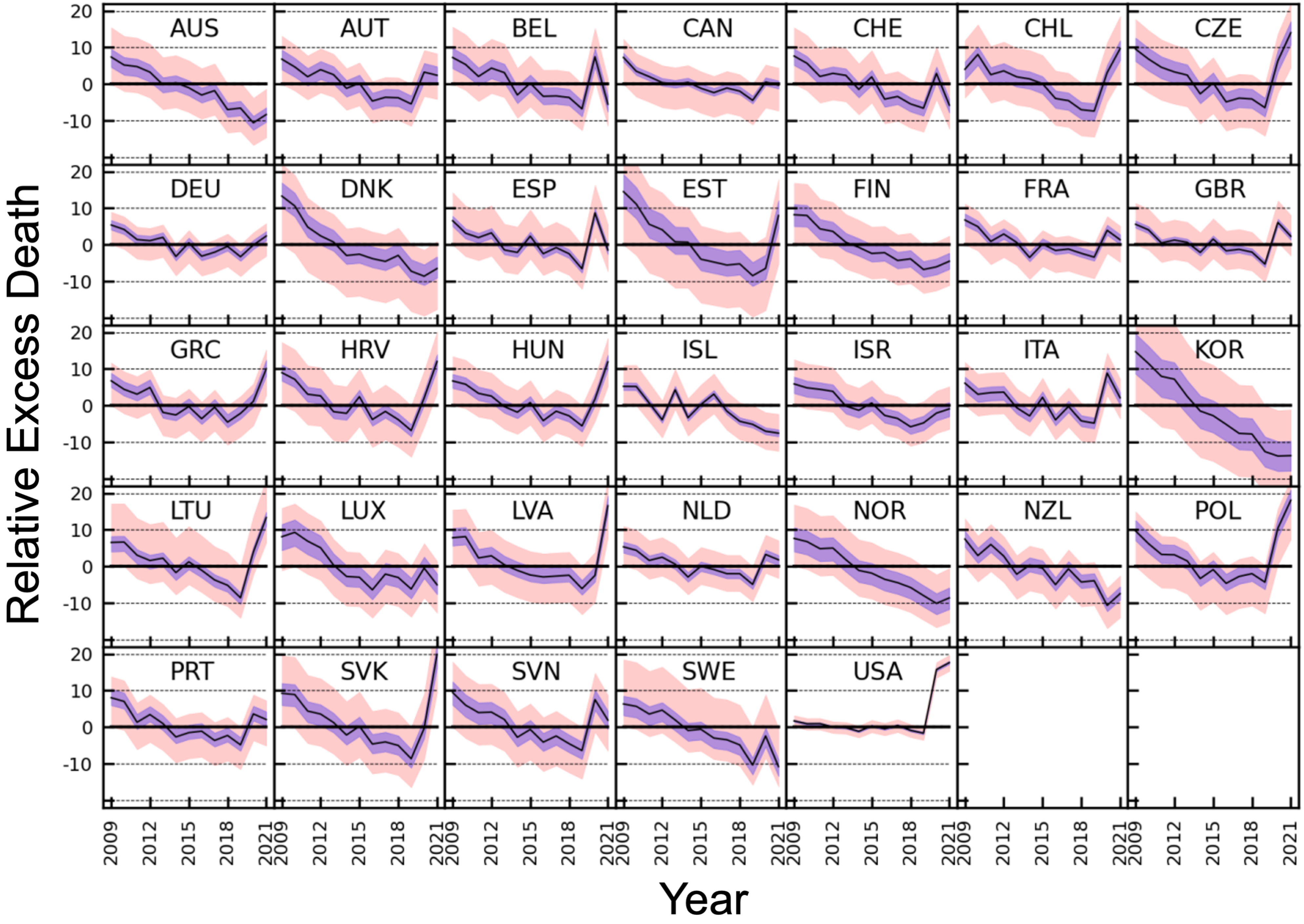

Figure S4B: Variation with year from 2009 to 2021 of the Relative Excess Death, p%, averaged over three adjacent years. The salmon shading marks the full range for all 66 reference sets, the purple shading marks the range between the first and third quartile and the black line shows the average over the reference periods. Note that the corresponding figure for averaging over two adjacent years is shown in main text **Figure 2**.

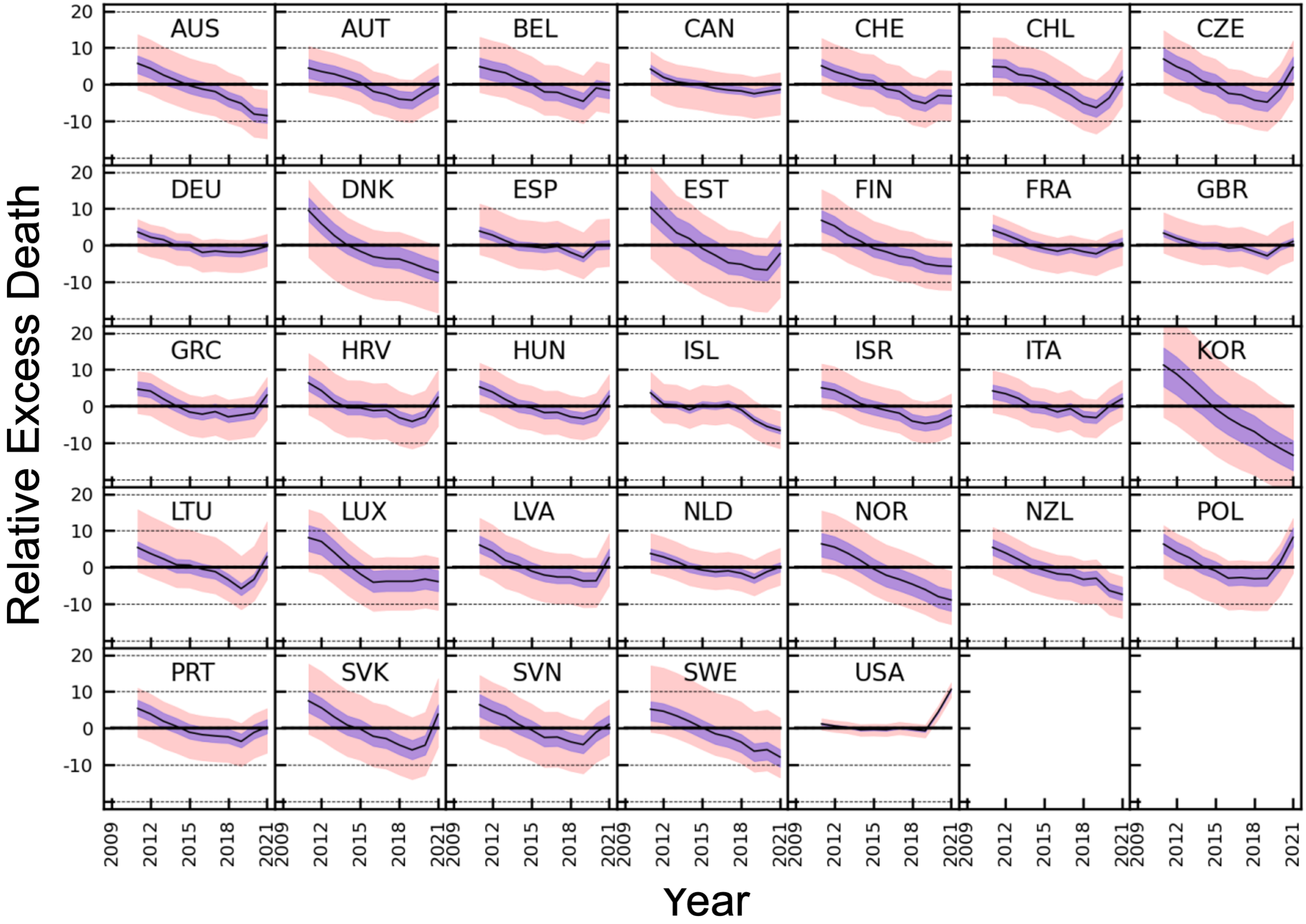

Figure S4C: Variation with year from 2009 to 2021 of the relative Excess Death p% averaged over four adjacent years. The salmon shading marks the full range for all 66 reference sets, the purple shading marks the range between the first and third quartile and the black line shows the average over the reference periods.

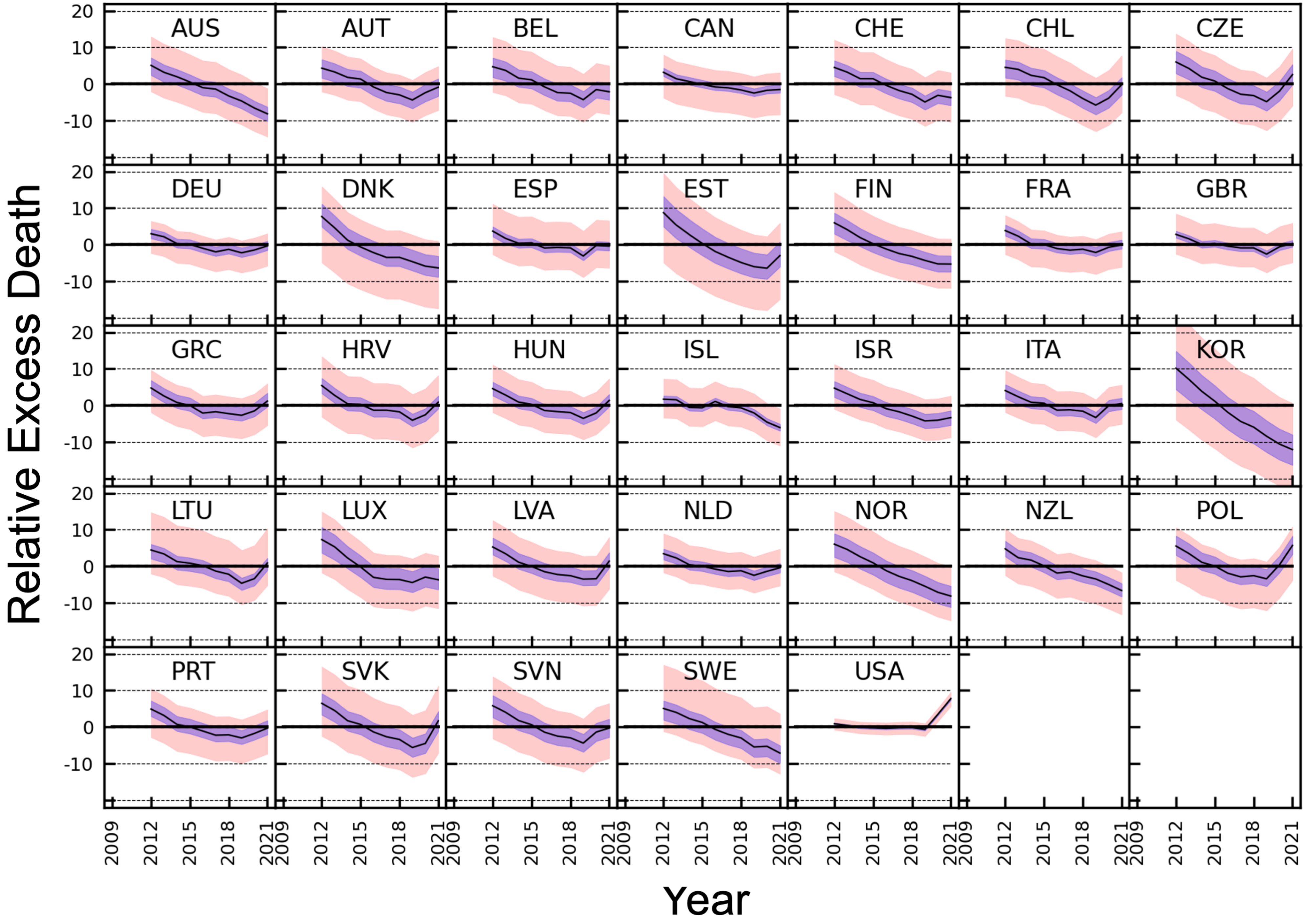

Table S1A: Relative percentage excess death, p%, in a single year for all countries and all years. Countries are listed in alphabetical order. Shading varies from solid green for the lowest values to solid red for the highest values. The shading is calibrated by range of values in all Tables S1 considered together. Note that 2019, the year immediately preceding the pandemics, is 'greenest' for all countries (low p%). By the same measure 2009 is 'reddest' (high p%).

Country	2009	2010	2011	2012	2013	2014	2015	2016	2017	2018	2019	2020	2021
Australia	7.36	5.18	4.76	3.24	-0.21	0.34	-1.03	-3.01	-1.81	-7.02	-6.60	-10.63	-8.36
Austria	6.78	4.66	1.99	3.91	2.54	-1.19	0.22	-4.67	-3.64	-3.77	-5.47	3.24	2.38
Belgium	7.23	5.34	2.06	4.33	3.10	-3.00	0.24	-3.37	-3.27	-3.69	-6.82	7.45	-5.54
Canada	7.25	3.49	2.00	0.39	-0.13	0.46	-1.17	-2.36	-1.10	-1.96	-4.48	0.53	-0.33
Chile	4.02	8.07	2.54	3.59	1.95	1.38	0.07	-3.97	-4.55	-7.06	-7.42	3.11	9.66
Croatia	8.98	7.17	3.09	2.63	-1.70	-2.15	2.41	-3.84	-1.63	-3.87	-6.86	2.03	12.04
Czechia	9.54	6.82	4.55	3.29	2.42	-2.70	0.30	-4.90	-3.87	-4.11	-6.49	6.36	14.03
Denmark	13.31	10.62	4.82	2.40	0.71	-2.97	-2.62	-3.82	-4.59	-2.94	-7.26	-8.63	-6.54
Estonia	14.52	11.13	5.58	4.07	0.77	0.67	-3.98	-4.77	-5.58	-5.26	-8.49	-6.51	7.99
Finland	8.21	8.01	4.32	3.62	0.64	-0.73	-2.40	-2.21	-4.33	-3.89	-6.80	-6.08	-4.54
France	6.76	4.93	0.89	2.90	0.61	-3.51	0.17	-1.60	-1.20	-2.37	-3.42	3.92	1.34
Germany	5.37	4.14	1.39	1.08	1.99	-3.25	0.50	-3.16	-2.07	-0.47	-3.31	-0.12	2.35
Greece	6.76	4.41	3.09	4.93	-1.89	-2.60	-0.28	-3.63	-0.48	-4.57	-2.16	1.12	10.06
Hungary	6.72	5.81	3.32	2.57	-0.32	-1.83	0.80	-4.15	-1.57	-2.84	-5.59	1.73	11.94
Iceland	5.23	5.20	0.67	-3.92	4.30	-3.34	0.26	3.13	-1.54	-4.32	-5.16	-7.05	-7.56
Israel	5.89	4.83	4.43	3.86	-0.17	-1.31	0.69	-2.72	-3.55	-5.83	-4.78	-2.09	-0.93
Italy	6.11	3.08	3.50	3.67	-0.60	-2.85	2.23	-3.95	-0.26	-4.22	-4.84	8.86	2.10
Latvia	7.84	8.13	2.31	2.88	0.45	-1.09	-2.36	-2.88	-2.65	-2.43	-6.11	-2.49	16.58
Lithuania	6.54	6.64	3.06	1.60	2.24	-1.71	1.24	-1.06	-3.71	-5.16	-8.61	3.90	13.35
Luxembourg	8.10	9.30	6.89	5.08	0.37	-2.75	-2.97	-6.44	-2.11	-3.04	-6.21	-0.48	-5.04
Netherlands	5.33	4.39	1.63	2.50	0.61	-2.92	0.03	-0.87	-1.99	-2.00	-4.94	3.25	1.79
New Zealand	7.46	2.95	5.99	2.81	-2.15	0.33	-0.49	-5.00	-0.67	-4.28	-3.92	-10.70	-7.48
Norway	7.64	6.77	4.84	5.01	1.82	-1.10	-1.85	-3.53	-4.46	-5.78	-8.00	-10.13	-8.63
Poland	9.69	6.22	3.27	3.17	1.48	-3.33	-0.88	-4.64	-2.76	-2.00	-4.32	10.43	18.09
Portugal	8.03	7.03	1.29	3.40	0.95	-2.71	-1.54	-1.13	-3.68	-2.31	-4.88	3.58	2.05
Slovakia	9.24	8.91	4.36	3.55	1.23	-2.18	0.23	-4.65	-4.08	-5.04	-8.67	-0.38	19.91
Slovenia	9.46	6.01	3.94	4.07	2.06	-2.80	-0.66	-4.14	-2.45	-4.56	-6.44	7.54	1.86
South Korea	14.72	11.48	7.96	7.20	2.47	-1.52	-2.79	-5.17	-7.62	-7.81	-12.61	-13.83	-13.72
Spain	6.58	3.15	1.93	3.22	-1.46	-2.13	2.32	-2.48	-0.81	-2.50	-6.59	8.70	-1.49
Sweden	6.33	5.68	3.54	4.62	1.95	-0.95	-0.62	-3.02	-3.48	-4.90	-10.38	-2.44	-10.83
Switzerland	7.63	5.65	2.06	2.90	2.36	-1.48	1.95	-4.15	-3.42	-5.47	-6.63	2.89	-5.82
United Kingdom	5.57	4.04	0.47	1.25	0.62	-2.13	1.52	-1.69	-1.29	-2.05	-5.32	6.09	2.27
United States	1.67	0.89	0.92	0.01	-0.20	-1.18	0.30	-0.50	0.43	-0.92	-1.69	15.73	17.65

Table S1B: Relative percentage excess death, p%, in two adjacent years for all countries and all years. When considering a two-year projected period, 2009 is added to 2010 and the combined period 2009+2010 is denoted as '2010'. For this reason, the entry for 2009 is marked as NA.

Country	2009	2010	2011	2012	2013	2014	2015	2016	2017	2018	2019	2020	2021
Australia	NA	6.25	4.97	3.98	1.49	0.07	-0.36	-2.04	-2.41	-4.45	-6.81	-8.64	-9.48
Austria	NA	5.71	3.32	2.96	3.22	0.66	-0.47	-2.25	-4.15	-3.71	-4.63	-1.10	2.81
Belgium	NA	6.27	3.68	3.21	3.71	0.02	-1.37	-1.58	-3.32	-3.48	-5.27	0.35	0.93
Canada	NA	5.31	2.73	1.18	0.12	0.17	-0.35	-1.78	-1.72	-1.53	-3.23	-1.94	0.09
Chile	NA	6.09	5.28	3.07	2.76	1.67	0.72	-2.01	-4.26	-5.82	-7.24	-2.08	6.43
Croatia	NA	8.07	5.11	2.85	0.45	-1.92	0.15	-0.74	-2.73	-2.76	-5.37	-2.40	7.04
Czechia	NA	8.16	5.67	3.92	2.86	-0.17	-1.19	-2.33	-4.38	-3.99	-5.31	-0.02	10.20
Denmark	NA	11.95	7.69	3.60	1.55	-1.15	-2.79	-3.22	-4.21	-3.76	-5.12	-7.95	-7.57
Estonia	NA	12.81	8.33	4.82	2.40	0.72	-1.68	-4.38	-5.17	-5.42	-6.88	-7.49	0.78
Finland	NA	8.11	6.14	3.97	2.11	-0.05	-1.58	-2.31	-3.28	-4.11	-5.36	-6.44	-5.30
France	NA	5.84	2.88	1.91	1.74	-1.48	-1.65	-0.72	-1.40	-1.79	-2.90	0.27	2.62
Germany	NA	4.75	2.75	1.23	1.54	-0.66	-1.35	-1.35	-2.61	-1.27	-1.90	-1.70	1.13
Greece	NA	5.56	3.74	4.02	1.47	-2.25	-1.42	-1.97	-2.04	-2.53	-3.36	-0.51	5.62
Hungary	NA	6.26	4.56	2.94	1.12	-1.08	-0.51	-1.69	-2.85	-2.21	-4.23	-1.91	6.84
Iceland	NA	5.22	2.91	-1.66	0.24	0.43	-1.52	1.71	0.77	-2.95	-4.75	-6.12	-7.31
Israel	NA	5.35	4.62	4.14	1.81	-0.75	-0.30	-1.03	-3.14	-4.71	-5.30	-3.42	-1.50
Italy	NA	4.56	3.30	3.59	1.51	-1.74	-0.29	-0.89	-2.09	-2.25	-4.53	2.05	5.45
Latvia	NA	7.98	5.21	2.59	1.66	-0.33	-1.73	-2.62	-2.77	-2.54	-4.27	-4.30	7.06
Lithuania	NA	6.59	4.84	2.32	1.92	0.26	-0.23	0.09	-2.39	-4.44	-6.89	-2.35	8.62
Luxembourg	NA	8.71	8.08	5.97	2.68	-1.22	-2.86	-4.73	-4.24	-2.58	-4.64	-3.32	-2.78
Netherlands	NA	4.86	2.99	2.07	1.55	-1.18	-1.43	-0.43	-1.44	-2.00	-3.48	-0.80	2.51
New Zealand	NA	5.16	4.49	4.38	0.30	-0.89	-0.08	-2.78	-2.80	-2.50	-4.10	-7.36	-9.07
Norway	NA	7.20	5.80	4.92	3.41	0.35	-1.48	-2.70	-4.00	-5.13	-6.90	-9.08	-9.37
Poland	NA	7.94	4.73	3.22	2.31	-0.96	-2.09	-2.78	-3.69	-2.38	-3.17	3.10	14.29
Portugal	NA	7.52	4.13	2.36	2.16	-0.90	-2.12	-1.34	-2.41	-2.99	-3.60	-0.62	2.80
Slovakia	NA	9.07	6.62	3.95	2.38	-0.50	-0.97	-2.24	-4.36	-4.56	-6.87	-4.48	9.86
Slovenia	NA	7.71	4.96	4.01	3.05	-0.40	-1.72	-2.42	-3.29	-3.52	-5.51	0.61	4.68
South Korea	NA	13.04	9.69	7.57	4.78	0.43	-2.17	-4.00	-6.42	-7.72	-10.26	-13.23	-13.77
Spain	NA	4.84	2.53	2.59	0.86	-1.80	0.11	-0.11	-1.64	-1.66	-4.57	1.11	3.59
Sweden	NA	6.00	4.61	4.08	3.27	0.49	-0.79	-1.83	-3.25	-4.19	-7.66	-6.37	-6.67
Switzerland	NA	6.63	3.84	2.49	2.63	0.42	0.25	-1.13	-3.78	-4.45	-6.06	-1.82	-1.50
United Kingdom	NA	4.79	2.24	0.86	0.94	-0.77	-0.30	-0.12	-1.49	-1.67	-3.70	0.43	4.17
United States	NA	1.27	0.91	0.46	-0.10	-0.70	-0.44	-0.10	-0.03	-0.25	-1.31	7.05	16.69

Table S1C: Relative percentage excess death, p%, in three adjacent years for all countries and all years. When considering three-year projected periods, 2009 and 2010 are added to 2011 and the combined period 2009+2010+2011 is denoted as '2011'. For this reason, the entries for both 2009 and 2010 are marked as NA.

Country	2009	2010	2011	2012	2013	2014	2015	2016	2017	2018	2019	2020	2021
Australia	NA	NA	5.73	4.37	2.55	1.09	-0.31	-1.27	-1.96	-3.98	-5.19	-8.12	-8.55
Austria	NA	NA	4.45	3.52	2.82	1.72	0.51	-1.91	-2.72	-4.02	-4.30	-1.98	0.07
Belgium	NA	NA	4.84	3.90	3.17	1.43	0.10	-2.05	-2.15	-3.45	-4.61	-0.98	-1.63
Canada	NA	NA	4.17	1.93	0.73	0.24	-0.28	-1.04	-1.55	-1.80	-2.54	-1.95	-1.39
Chile	NA	NA	4.87	4.70	2.69	2.29	1.13	-0.91	-2.89	-5.23	-6.37	-3.69	1.95
Croatia	NA	NA	6.38	4.27	1.31	-0.43	-0.46	-1.20	-1.04	-3.11	-4.14	-2.88	2.44
Czechia	NA	NA	6.93	4.86	3.41	0.97	-0.01	-2.45	-2.85	-4.29	-4.84	-1.36	4.70
Denmark	NA	NA	9.53	5.89	2.62	0.01	-1.65	-3.14	-3.69	-3.78	-4.95	-6.32	-7.47
Estonia	NA	NA	10.37	6.88	3.44	1.81	-0.88	-2.73	-4.78	-5.20	-6.45	-6.76	-2.28
Finland	NA	NA	6.81	5.28	2.83	1.14	-0.86	-1.79	-2.99	-3.49	-5.02	-5.60	-5.79
France	NA	NA	4.15	2.89	1.47	-0.05	-0.91	-1.63	-0.88	-1.73	-2.34	-0.60	0.63
Germany	NA	NA	3.61	2.18	1.49	-0.09	-0.26	-1.97	-1.59	-1.89	-1.96	-1.30	-0.33
Greece	NA	NA	4.70	4.15	2.00	0.08	-1.57	-2.17	-1.46	-2.89	-2.41	-1.85	3.06
Hungary	NA	NA	5.27	3.89	1.84	0.12	-0.44	-1.74	-1.65	-2.85	-3.35	-2.22	2.73
Iceland	NA	NA	3.67	0.58	0.38	-0.98	0.38	0.07	0.60	-0.97	-3.71	-5.53	-6.61
Israel	NA	NA	5.03	4.36	2.65	0.74	-0.26	-1.12	-1.89	-4.06	-4.73	-4.20	-2.56
Italy	NA	NA	4.19	3.42	2.16	0.02	-0.39	-1.53	-0.68	-2.81	-3.12	-0.01	2.07
Latvia	NA	NA	6.08	4.43	1.87	0.73	-1.01	-2.12	-2.63	-2.65	-3.73	-3.68	2.68
Lithuania	NA	NA	5.41	3.75	2.30	0.70	0.59	-0.50	-1.18	-3.31	-5.83	-3.28	2.89
Luxembourg	NA	NA	8.09	7.05	4.04	0.80	-1.83	-4.09	-3.83	-3.83	-3.82	-3.23	-3.90
Netherlands	NA	NA	3.75	2.83	1.57	0.02	-0.77	-1.24	-0.96	-1.63	-3.00	-1.19	0.08
New Zealand	NA	NA	5.45	3.92	2.15	0.31	-0.75	-1.77	-2.05	-3.31	-2.98	-6.36	-7.40
Norway	NA	NA	6.40	5.53	3.88	1.88	-0.39	-2.17	-3.29	-4.60	-6.10	-8.00	-8.93
Poland	NA	NA	6.35	4.20	2.62	0.39	-0.93	-2.96	-2.78	-3.12	-3.03	1.43	8.18
Portugal	NA	NA	5.39	3.88	1.88	0.50	-1.12	-1.79	-2.13	-2.38	-3.63	-1.17	0.29
Slovakia	NA	NA	7.48	5.58	3.03	0.83	-0.25	-2.22	-2.86	-4.59	-5.96	-4.67	3.80
Slovenia	NA	NA	6.42	4.66	3.34	1.05	-0.49	-2.55	-2.43	-3.72	-4.52	-1.08	1.04
South Korea	NA	NA	11.26	8.83	5.80	2.59	-0.69	-3.21	-5.26	-6.91	-9.42	-11.50	-13.40
Spain	NA	NA	3.84	2.77	1.21	-0.16	-0.40	-0.77	-0.35	-1.93	-3.34	-0.07	0.24
Sweden	NA	NA	5.17	4.61	3.36	1.85	0.11	-1.54	-2.39	-3.81	-6.29	-5.89	-7.89
Switzerland	NA	NA	5.07	3.52	2.44	1.23	0.94	-1.25	-1.91	-4.35	-5.20	-3.01	-3.18
United Kingdom	NA	NA	3.31	1.90	0.78	-0.11	0.00	-0.78	-0.52	-1.68	-2.91	-0.39	1.05
United States	NA	NA	1.15	0.60	0.24	-0.47	-0.36	-0.46	0.08	-0.33	-0.74	4.42	10.60

Table S1D: Percentage excess death, p%, in four adjacent years for all countries and all years. When considering four-year projected periods, 2009, 2010 and 2011 are added to 2012 and the combined period 2009+2010+2011+2012 is denoted as '2012'. For this reason, the entries for both 2009, 2010 and 2011 are marked as NA. With a longer projected period, the relative percentage excess death gets smaller for every country.

Country	2009	2010	2011	2012	2013	2014	2015	2016	2017	2018	2019	2020	2021
**Australia**	NA	NA	NA	5.08	3.17	1.97	0.54	-1.02	-1.41	-3.27	-4.66	-6.61	-8.18
**Austria**	NA	NA	NA	4.31	3.27	1.79	1.33	-0.83	-2.35	-2.99	-4.39	-2.39	-0.88
**Belgium**	NA	NA	NA	4.71	3.70	1.58	1.13	-0.79	-2.36	-2.54	-4.31	-1.54	-2.14
**Canada**	NA	NA	NA	3.17	1.39	0.66	-0.12	-0.82	-1.06	-1.65	-2.50	-1.74	-1.53
**Chile**	NA	NA	NA	4.54	3.98	2.35	1.72	-0.22	-1.87	-3.99	-5.80	-3.89	-0.21
**Croatia**	NA	NA	NA	5.42	2.74	0.42	0.29	-1.32	-1.31	-1.76	-4.07	-2.57	0.88
**Czechia**	NA	NA	NA	6.00	4.24	1.84	0.79	-1.27	-2.82	-3.18	-4.86	-1.97	2.54
**Denmark**	NA	NA	NA	7.70	4.56	1.18	-0.66	-2.20	-3.51	-3.49	-4.67	-5.90	-6.38
**Estonia**	NA	NA	NA	8.75	5.30	2.72	0.31	-1.89	-3.46	-4.91	-6.04	-6.47	-3.01
**Finland**	NA	NA	NA	5.98	4.07	1.90	0.22	-1.21	-2.45	-3.22	-4.33	-5.29	-5.33
**France**	NA	NA	NA	3. 82	2.30	0.18	0.01	-1.09	-1.52	-1.27	-2.16	-0.74	-0.10
**Germany**	NA	NA	NA	2.96	2.13	0.27	0.06	-1.01	-1.99	-1.31	-2.25	-1.49	-0.36
**Greece**	NA	NA	NA	4.76	2.57	0.80	-0.02	-2.11	-1.74	-2.25	-2.71	-1.51	1.18
**Hungary**	NA	NA	NA	4.59	2.82	0.91	0.30	-1.39	-1.70	-1.95	-3.55	-2.06	1.36
**Iceland**	NA	NA	NA	1.71	1.54	-0.59	-0.66	1.09	-0.35	-0.68	-2.06	-4.57	-6.06
**Israel**	NA	NA	NA	4.73	3.17	1.62	0.73	-0.90	-1.75	-2.91	-4.24	-4.05	-3.35
**Italy**	NA	NA	NA	4.06	2.38	0.86	0.59	-1.30	-1.21	-1.58	-3.33	-0.07	0.53
**Latvia**	NA	NA	NA	5.27	3.42	1.12	-0.06	-1.49	-2.25	-2.58	-3.52	-3.42	1.40
**Lithuania**	NA	NA	NA	4.44	3.37	1.28	0.84	0.17	-1.31	-2.18	-4.65	-3.39	0.88
**Luxembourg**	NA	NA	NA	7.30	5.30	2.25	-0.19	-3.04	-3.58	-3.63	-4.45	-2.96	-3.69
**Netherlands**	NA	NA	NA	3.43	2.25	0.41	0.02	-0.79	-1.44	-1.23	-2.48	-1.39	-0.42
**New Zealand**	NA	NA	NA	4.76	2.34	1.67	0.10	-1.86	-1.48	-2.63	-3.47	-4.99	-6.65
**Norway**	NA	NA	NA	6.05	4.59	2.61	0.93	-1.19	-2.76	-3.93	-5.47	-7.14	-8.16
**Poland**	NA	NA	NA	5.53	3.49	1.08	0.06	-1.89	-2.91	-2.58	-3.43	0.40	5.69
**Portugal**	NA	NA	NA	4.88	3.12	0.70	-0.02	-1.12	-2.27	-2.17	-3.02	-1.78	-0.34
**Slovakia**	NA	NA	NA	6.48	4.47	1.69	0.67	-1.39	-2.70	-3.43	-5.64	-4.52	1.66
**Slovenia**	NA	NA	NA	5.81	3.98	1.74	0.60	-1.44	-2.52	-2.99	-4.42	-1.41	-0.32
**South Korea**	NA	NA	NA	10.17	7.13	3.84	1.15	-1.88	-4.39	-5.94	-8.43	-10.59	-12.09
**Spain**	NA	NA	NA	3.68	1.68	0.35	0.48	-0.94	-0.78	-0.90	-3.13	-0.25	-0.43
**Sweden**	NA	NA	NA	5.03	3.93	2.26	1.22	-0.69	-2.04	-3.03	-5.49	-5.30	-7.16
**Switzerland**	NA	NA	NA	4.51	3.22	1.43	1.41	-0.38	-1.81	-2.83	-4.94	-3.11	-3.73
**United Kingdom**	NA	NA	NA	2.78	1.57	0.03	0.31	-0.44	-0.91	-0.91	-2.61	-0.61	0.29
**United States**	NA	NA	NA	0.86	0.40	-0.13	-0.27	-0.39	-0.23	-0.18	-0.68	3.45	7.77

Table S2: The percentage excess death , p%, of the worst, the second worst and third worst pairs of adjacent years compared to the corresponding value in two-year pandemic projected period 2020+2021. Those entries where the two-year pandemic period 2020+2021 are worst are shown in bold type.

Country	Worst Pair Years	2nd Worst Pair Years	3rd Worst Pair Years	Worst p%	2nd Worst p%	3rd Worst p%	2020+2021 p%	Worst minus (2020+2021) p%
**Australia**	2009+2010	2010+2011	2011+2012	6.2	5.0	4.0	-9.5	15.7
**Austria**	2009+2010	2010+2011	2012+2013	5.7	3.3	3.2	2.8	2.9
**Belgium**	2009+2010	2012+2013	2010+2011	6.3	3.7	3.7	0.9	5.3
**Canada**	2009+2010	2010+2011	2011+2012	5.3	2.7	1.2	0.1	5.2
**Switzerland**	2009+2010	2010+2011	2012+2013	6.6	3.8	2.6	-1.5	8.1
**Chile**	**2020+2021**	2009+2010	2010+2011	**6.4**	6.1	5.3	6.4	0.0
**Czechia**	**2020+2021**	2009+2010	2010+2011	**10.2**	8.2	5.7	10.2	0.0
**Germany**	2009+2010	2010+2011	2012+2013	4.8	2.8	1.5	1.1	3.6
**Denmark**	2009+2010	2010+2011	2011+2012	12.0	7.7	3.6	-7.6	19.5
**Spain**	2009+2010	**2020+2021**	2011+2012	4.8	**3.6**	2.6	3.6	1.3
**Estonia**	2009+2010	2010+2011	2011+2012	12.8	8.3	4.8	0.8	12.0
**Finland**	2009+2010	2010+2011	2011+2012	8.1	6.1	4.0	-5.3	13.4
**France**	2009+2010	2010+2011	**2020+2021**	5.8	2.9	**2.6**	2.6	3.2
**United Kingdom**	2009+2010	**2020+2021**	2010+2011	4.8	**4.2**	2.2	4.2	0.6
**Greece**	**2020+2021**	2009+2010	2011+2012	**5.6**	5.6	4.0	5.6	0.0
**Croatia**	2009+2010	**2020+2021**	2010+2011	8.1	**7.0**	5.1	7.0	1.0
**Hungary**	**2020+2021**	2009+2010	2010+2011	**6.8**	6.3	4.6	6.8	0.0
**Iceland**	2009+2010	2010+2011	2015+2016	5.2	2.9	1.7	-7.3	12.5
**Israel**	2009+2010	2010+2011	2011+2012	5.4	4.6	4.1	-1.5	6.9
**Italy**	**2020+2021**	2009+2010	2011+2012	**5.5**	4.6	3.6	5.5	0.0
**South Korea**	2009+2010	2010+2011	2011+2012	13.0	9.7	7.6	-13.8	26.8
**Lithuania**	**2020+2021**	2009+2010	2010+2011	**8.6**	6.6	4.8	8.6	0.0
**Luxembourg**	2009+2010	2010+2011	2011+2012	8.7	8.1	6.0	-2.8	11.5
**Latvia**	2009+2010	**2020+2021**	2010+2011	8.0	**7.1**	5.2	7.1	0.9
**Netherlands**	2009+2010	2010+2011	**2020+2021**	4.9	3.0	**2.5**	2.5	2.3
**Norway**	2009+2010	2010+2011	2011+2012	7.2	5.8	4.9	-9.4	16.6
**New Zealand**	2009+2010	2010+2011	2011+2012	5.2	4.5	4.4	-9.1	14.2
**Poland**	**2020+2021**	2009+2010	2010+2011	**14.3**	7.9	4.7	14.3	0.0
**Portugal**	2009+2010	2010+2011	**2020+2021**	7.5	4.1	**2.8**	2.8	4.7
**Slovakia**	**2020+2021**	2009+2010	2010+2011	9.9	9.1	6.6	9.9	0.0
**Slovenia**	2009+2010	2010+2011	**2020+2021**	**7.7**	5.0	**4.7**	4.7	3.0
**Sweden**	2009+2010	2010+2011	2011+2012	6.0	4.6	4.1	-6.7	12.7
**United States**	**2020+2021**	2019+2020	2009+2010	**16.7**	7.0	1.3	16.7	0.0

Table S3: Supplementary links to data sources.

Country	LOC	Death File Name	Last Modification Date	Population File Name	Last Modification Date	Death Download Link	Population Download Link
All HMD Short Term Mortality Fluctuations		stmf.csv	20-May-2022	stmf.csv	20-May-2022	https://www.mortality.org/File/GetDocument/Public/STMF/Outputs/stmf.csv	
Australia	AUS	Australia_Deaths_1x1.txt	22-Mar-2022	Australia_Population.txt	22-Mar-2022	AUS.Deaths_1x1.txt	AUS.Population.txt
Austria	AUT	Austria_Deaths_1x1.txt	30-Mar-2021	Austria_Population.txt	30-Mar-2021	AUT.Deaths_1x1.txt	AUT.Population.txt
Belgium	BEL	Belgium_Deaths_1x1.txt	25-Sep-2021	Belgium_Population.txt	25-Sep-2021	BEL.Deaths_1x1.txt	BEL.Population.txt
Canada	CAN	Canada_Deaths_1x1.txt	28-Sep-2021	Canada_Population.txt	28-Sep-2021	CAN.Deaths_1x1.txt	CAN.Population.txt
Switzerland	CHE	Switzerland_Deaths_1x1.txt	28-Oct-2021	Switzerland_Population.txt	28-Oct-2021	CHE.Deaths_1x1.txt	CHE.Population.txt
Chile	CHL	Chile_Deaths_1x1.txt	18-Apr-2022	Chile_Population.txt	18-Apr-2022	CHL.Deaths_1x1.txt	CHL.Population.txt
Czechia	CZE	Czechia_Deaths_1x1.txt	23-May-2021	Czechia_Population.txt	23-May-2021	CZE.Deaths_1x1.txt	CZE.Population.txt
Germany	DEU	Germany_Deaths_1x1.txt	17-Dec-2018	Germany_Population.txt	17-Dec-2018	DEUTNP.Deaths_1x1.txt	DEUTNP.Population.txt
Denmark	DNK	Denmark_Deaths_1x1.txt	22-Mar-2022	Denmark_Population.txt	22-Mar-2022	DNK.Deaths_1x1.txt	DNK.Population.txt
Spain	ESP	Spain_Deaths_1x1.txt	23-Feb-2022	Spain_Population.txt	23-Feb-2022	ESP.Deaths_1x1.txt	ESP.Population.txt
Estonia	EST	Estonia_Deaths_1x1.txt	21-Jan-2021	Estonia_Population.txt	21-Jan-2021	EST.Deaths_1x1.txt	EST.Population.txt
Finland	FIN	Finland_Deaths_1x1.txt	02-Aug-2021	Finland_Population.txt	02-Aug-2021	FIN.Deaths_1x1.txt	FIN.Population.txt
France	FRA	France_Deaths_1x1.txt	11-Apr-2022	France_Population.txt	11-Apr-2022	FRATNP.Deaths_1x1.txt	FRATNP.Population.txt
United Kingdom	GBR	UK_Deaths_1x1.txt	11-Jul-2020	UK_Population.txt	11-Jul-2020	GBR_NP.Deaths_1x1.txt	GBR_NP.Population.txt
Greece	GRC	Greece_Deaths_1x1.txt	08-Nov-2021	Greece_Population.txt	08-Nov-2021	GRC.Deaths_1x1.txt	GRC.Population.txt
Croatia	HRV	Croatia_Deaths_1x1.txt	24-Feb-2022	Croatia_Population.txt	24-Feb-2022	HRV.Deaths_1x1.txt	HRV.Population.txt
Hungary	HUN	Hungary_Deaths_1x1.txt	30-Nov-2021	Hungary_Population.txt	30-Nov-2021	HUN.Deaths_1x1.txt	HUN.Population.txt
Iceland	ISL	Iceland_Deaths_1x1.txt	02-Apr-2020	Iceland_Population.txt	02-Apr-2020	ISL.Deaths_1x1.txt	ISL.Population.txt
Israel	ISR	Israel_Deaths_1x1.txt	31-Oct-2018	Israel_Population.txt	31-Oct-2018	ISR.Deaths_1x1.txt	ISR.Population.txt
Italy	ITA	Italy_Deaths_1x1.txt	11-Apr-2022	Italy_Population.txt	11-Apr-2022	ITA.Deaths_1x1.txt	ITA.Population.txt
South Korea	KOR	Republic_of_Korea_Deaths_1x1.txt	15-Nov-2019	Republic_of_Korea_Population.txt	15-Nov-2019	KOR.Deaths_1x1.txt	KOR.Population.txt
Lithuania	LTU	Lithuania_Deaths_1x1.txt	29-Jan-2022	Lithuania_Population.txt	29-Jan-2022	LTU.Deaths_1x1.txt	LTU.Population.txt
Luxembourg	LUX	Luxembourg_Deaths_1x1.txt	21-Jan-2022	Luxembourg_Population.txt	21-Jan-2022	LUX.Deaths_1x1.txt	LUX.Population.txt
Latvia	LVA	Latvia_Deaths_1x1.txt	11-Mar-2021	Latvia_Population.txt	11-Mar-2021	LVA.Deaths_1x1.txt	LVA.Population.txt
Netherlands	NLD	Netherlands_Deaths_1x1.txt	31-Mar-2021	Netherlands_Population.txt	31-Mar-2021	NLD.Deaths_1x1.txt	NLD.Population.txt
Norway	NOR	Norway_Deaths_1x1.txt	15-Apr-2021	Norway_Population.txt	15-Apr-2021	NOR.Deaths_1x1.txt	NOR.Population.txt
New Zealand	NZL	New_Zealand_Deaths_1x1.txt	26-Sep-2017	New_Zealand_Population.txt	26-Sep-2017	NZL_NP.Deaths_1x1.txt	NZL_NP.Population.txt
Poland	POL	Poland_Deaths_1x1.txt	13-Apr-2021	Poland_Population.txt	13-Apr-2021	POL.Deaths_1x1.txt	POL.Population.txt
Portugal	PRT	Portugal_Deaths_1x1.txt	01-Aug-2021	Portugal_Population.txt	01-Aug-2021	PRT.Deaths_1x1.txt	PRT.Population.txt
Slovakia	SVK	Slovakia_Deaths_1x1.txt	29-Oct-2021	Slovakia_Population.txt	29-Oct-2021	SVK.Deaths_1x1.txt	SVK.Population.txt
Slovenia	SVN	Slovenia_Deaths_1x1.txt	01-Nov-2021	Slovenia_Population.txt	01-Nov-2021	SVN.Deaths_1x1.txt	SVN.Population.txt
Sweden	SWE	Sweden_Deaths_1x1.txt	12-May-2022	Sweden_Population.txt	12-May-2022	SWE.Deaths_1x1.txt	SWE.Population.txt
United States	USA	USA_Deaths_1x1.txt	17-Mar-2021	USA_Population.txt	17-Mar-2021	USA.Deaths_1x1.txt	USA.Population.txt

All Deaths_1x1.txt and Population.txt files are downloaded as a zip file **https://www.mortality.org/File/Download/hmd.v6/zip/all_hmd/hmd_statistics_20220812.zip**, where the Version shown here is "hmd_statistics_20220812" and changes frequently. The relevant files for Deaths and Population are in directories **/<Version>/deaths/Deaths_1x1/** and **/<Version>/population/Population/** respectively. More generally, the download button for the latest version is marked "All HMD Statistics" and is at the bottom of the page linked by **https://www.mortality.org/Data/ZippedDataFiles**
